# Glomerular C4 deposition and glomerulosclerosis predict worse renal outcomes in Chinese patients with IgA nephropathy

**DOI:** 10.1080/0886022X.2020.1786400

**Published:** 2020-07-14

**Authors:** Yan Yang, Xi Tang, Yuan Yang, Xinrui Li, Lingzhi Li, Kai Huang, Yi Li, Jing Li, Ping Fu

**Affiliations:** a Department of Nephrology, The Third Affiliated Hospital of Soochow University, Changzhou, P.R. China; b Division of Nephrology, West China Hospital, Kidney Research Institute, Sichuan University, Chengdu, P.R. China; c Department of Biostatistics, School of Public Health, University of Michigan, Ann Arbor, MI, USA; d Department of Orthopaedic Surgery, West China Hospital, Sichuan University, Chengdu, P.R. China; e Department of Nephrology, The First Affiliated Hospital of Xinjiang Medical University, Urumqi, P.R. China

**Keywords:** C4 deposition, IgA nephropathy, glomerulosclerosis

## Abstract

**Background:**

Immunoglobulin A nephropathy (IgAN) is a clinical and pathological syndrome with heterogenous manifestation and progression. Complement activation is involved in the disease. However, the clinical significance of C4 deposition in IgAN is obscure.

**Methods:**

A multicenter retrospective study was conducted in biopsy-proven IgAN patients. Based on mesangial C4 deposition, patients were divided into two groups. The baseline clinical data and immunopathological phenotypes were compared. The composite endpoint was defined as eGFR decline greater than 50%, doubling of baseline serum creatinine, the occurrence of end-stage renal disease (ESRD).

**Results:**

A total of 642 IgAN patients were recruited, with 41 patients showing mesangial C4 deposition. The mesangial C4 positive group showed lower serum albumin, higher proteinuria, and a higher rate of IgG, IgM, and C1q mesangial deposition. After a median follow-up of 43.18 months, 81 (12.62%) patients achieved the composite endpoint. The multivariate Cox regression models identified glomerular C4 deposition (hazard ratios [HR] = 3.22, 95% confidence intervals [CI] = 1.51–6.87, *p* < 0.01), global sclerosis (G1 vs. G0, HR = 1.90, 95%CI = 1.02–3.52, *p* = 0.04; G2 vs. G0, HR = 3.72, 95%CI = 1.98–7.00, *p* < 0.01), male (HR = 1.80, 95%CI = 1.10–2.97, *p* = 0.02), serum creatinine (HR = 1.01, 95%CI = 1.00–1.01, *p* < 0.01), triglyceride (HR = 1.17, 95%CI = 1.01–1.35, *p* = 0.04), proteinuria (HR = 1.07, 95%CI = 1.01–1.13, *p* = 0.02), serum C3 level (HR = 0.05, 95%CI = 0.01–0.25, *p* < 0.01), and serum C4 level (HR = 99.59, 95%CI = 8.69–1140.89, *p* < 0.01) as independent risk factors for poor renal outcomes.

**Conclusions:**

Glomerular mesangial C4 deposition and global sclerosis are independent predictors for poor prognosis in IgAN patients.

## Introduction

Immunoglobulin A nephropathy (IgAN) is regarded as the most common primary glomerulonephritis worldwide, especially in Asia [1]. IgAN patients present a variety of clinical manifestations including isolated hematuria, nephrotic syndrome, and rapidly progressive glomerulonephritis. The histopathological features occur in varied forms as well. In recent years, studies with long-term follow-up found that IgAN is related to poor renal outcomes [2,3], and about 30%∼40% of IgAN patients progressed to end-stage renal disease (ESRD) within 10 ∼ 25 years [4].

Previous studies identified renal impairment at renal biopsy, severe histologic grading, and proteinuria as predictors of IgAN prognosis [2,5,6]. The Oxford Classification of IgAN updated and incorporated cellular or fibrocellular crescents as the C-score in addition to the MEST score [7]. However, the association between crescents and the prognosis of IgAN is still controversial. In addition, the immune morphology is not included in the histological classification of IgAN.

IgAN is considered as an autoimmune-mediated inflammatory disease [8]. Immune complexes deposition in the glomerular mesangium leads to the activation of complement pathways and initiation of immune-mediated inflammation. The activation of alternative and lectin complement pathways plays an important role in the pathogenesis and progression of IgAN [9,10]. Complement components and complement regulatory factors in renal tissue, urine, and serum samples may be useful biomarkers to evaluate the activation of the complement system and predict the prognosis of IgAN [9]. Several prior studies have determined that circulating complement levels or complement deposition is correlated to renal survival [11,12]. Specifically, mesangial C3 and C4d deposition could predict renal outcomes in IgAN patients [12,13]. C4 participated in the activation of complement through classical and lectin pathways [9]. However, the correlation between C4 staining and IgAN prognosis remains unclear. In this study, we evaluated the predictive value of glomerular C4 deposition and widely accepted clinicopathological features, aiming to conduct a comprehensive prognosis study to assist physicians in predicting renal outcomes for IgAN patients and providing effective treatments.

## Materials and methods

### Study design and population

We conducted a multicenter retrospective study in biopsy-proven IgAN patients admitted to the West China Hospital of Sichuan University and First Affiliated Hospital of Xinjiang Medical University between January 2011 and December 2014. Patients with a follow-up time of more than 1 year were enrolled. Those who reached the composite endpoint within 1 year were also included. The deposits limited to the mesangium in at least one nonsclerotic glomerulus were recorded in this study. The exclusion criteria included: (1) fewer than eight glomeruli in a renal tissue section for diagnosis; (2) secondary IgA deposition caused by purpura, chronic hepatitis, systemic lupus erythematosus, and others; (3) incidence of malignant tumor, acute severe infection and serious cardio-cerebrovascular diseases; (4) renal transplant recipients; (5) age <18 years. This study was conducted following the principles of the Declaration of Helsinki and with the approval of the Ethics Committee at the West China Hospital of Sichuan University and the First Affiliated Hospital of Xinjiang Medical University. Written informed consent was obtained from all patients.

### Data collections

Demographics, such as gender, age, and nationality, were collected upon admission. Clinical data including urine red blood cell counts, 24 h urinary protein, hemoglobin, serum creatine, albumin, uric acid, triglyceride, and serum complement level were obtained within 1 week of renal biopsy. Estimated glomerular filtration rate (eGFR) was calculated using the Chronic Kidney Disease Epidemiology Collaboration (CKD-EPI) equation [14]. Hypertension status (blood pressure (BP) >140/90 mmHg or receiving antihypertensive drugs) was recorded. Mean arterial pressure (MAP) was defined as diastolic BP plus 1/3 of the pulse pressure. Comprehensive supportive care including angiotensin-converting enzyme inhibitor (ACEI) or angiotensin receptor blockers (ARB) were given to patients to achieve a target BP below 125/75 mmHg. Patients also received the immunosuppressive therapy (corticosteroids, cyclophosphamide, mycophenolate mofetil, or other immunosuppressive agents) in addition to supportive care according to clinical and pathological severity.

Histological sections were reviewed by two experienced renal pathologists (Huan Xu and Lin Li), who were blinded to the clinical data and renal outcomes, based on the updated Oxford Classification criteria for IgAN [7]. Furthermore, global sclerosis (G) was defined as scarring lesion or hyaline deposition appearing in at least 50% of one glomerulus. G0, G1, and G2 were defined by the percentage of global sclerosis (0–25%, 26%–50%, and >50% of glomeruli, respectively). An equation (age/2–10) was used to determine the proportion of sclerotic glomeruli as pathological sclerosis [15]. Direct immunofluorescence for IgG, IgA, IgM, C1q, C3, C4, λ, κ, and fibrin were routinely performed on 5-mm frozen sections. C4 was stained using rabbit anti-human C4c (OriGene, Beijing, China). Each specimen was scored using digital images and a real-time scoring system (Olympus BX41, Japan). The intensity of immune complex deposition in glomeruli or mesangial area was rated from 0− to 3+++. Patients were divided according to glomerular C4 deposition status: the C4 negative group and the C4 positive group.

### Outcomes

The composite endpoint was defined as eGFR decline >50%, doubling of baseline serum creatinine, the occurrence of ESRD, where ESRD contained eGFR <15 ml/min/1.73 m^2^, dialysis, and kidney transplantation.

### Statistical analyses

Continuous variables with normal distribution were compared by Student’s *t*-test and the results were presented as mean ± standard deviation. Non-normal distribution variables were compared by non-parametric test (Mann–Whitney *U* test or Kruskal–Wallis *H* test) and the results were presented as median (25th to 75th percentiles). Categorical or ordinal variables were compared by Pearson Chi-Square test, Fisher’s exact test, or Wilcoxon rank sum test. The results were presented as frequencies (percentages). The prognostic value of immune staining and global sclerosis was analyzed by Kaplan–Meier method. To identify the independent risk factors, we performed univariate Cox regression models. When *p*-value is less than 0.05, the variables will be included in multivariable Cox regression models using the ‘Forward’ method. Hazard ratios (HR) and 95% confidence intervals (CI) were provided. All statistical analyses were conducted in SPSS 24.0 software. The statistical significance level was set at *p* < 0.05 and all the statistical tests were two-sided.

## Results

### Clinical parameters

A total of 901 patients with primary biopsy-proven IgAN were enrolled according to the exclusion criteria, and 642 patients were followed up and available for final analysis. The flowchart of the patients’ enrollment was shown in Figure S1. Among the 642 patients, 281 (43.77%) of them were male. The median age at IgAN diagnosis was 33.23 years. There were 222 (34.60%) patients having hypertension as a comorbidity. The majority of patients received comprehensive supportive care, and more than 50% received immunosuppressive therapy. Forty-one patients (6.39%) showed C4 deposition and were classified into the C4 positive group. Table 1 shows the baseline clinical characteristics of patients at the time of renal biopsy. Patients in the C4 positive group showed lower serum albumin and higher proteinuria (*p* < 0.05). There were no significant differences in age, serum creatinine, uric acid, and serum C3 and C4 level between the C4 negative and positive groups.

**Table 1.** Baseline clinical characteristics and histopathologic features of 642 IgAN patients at the time of renal biopsy.

Characteristics	Total (*N* = 642)	C4 negative group (*n* = 601)	C4 positive group (*n* = 41)	*p*-value
Male (*n*, %)	281 (43.77%)	267 (44.43%)	14 (34.15%)	0.20
Age (years)	33.23 (26.83, 42.50)	33.23 (26.53, 42.39)	34.00 (29.00, 44.92)	0.17
MAP (mmHg)	97.00 (89.00, 108.00)	97.00 (88.67, 107.67)	101.50 (91.17, 117.83)	0.15
Hypertension (*n*, %)	222 (34.60%)	205 (34.10%)	17 (41.50%)	0.29
Hemoglobin (g/L)	133.00 (122.00, 148.00)	133.00 (122.00, 149.00)	133.00 (122.00, 142.00)	0.54
Albumin (g/L)	39.40 (35.50, 42.80)	39.50 (35.70, 43.00)	36.30 (32.10, 40.60)	<0.01
Serum creatinine (µmol/L)	86.35 (67.00, 118.10)	86.60 (67.00, 117.20)	82.84 (63.80, 137.00)	0.97
eGFR (ml/min/1.73m^2^)	85.84 (60.90, 110.30)	85.87 (61.59, 109.91)	85.05 (48.61, 110.50)	0.46
Uric acid (µmol/L)	366.35 (300.70, 442.00)	365.70 (300.70, 442.00)	385.20 (303.30, 426.70)	0.93
Triglyceride (mmol/L)	1.52 (1.05, 2.27)	1.51 (1.04, 2.24)	1.88 (1.18, 3.20)	0.07
Cholesterol (mmol/L)	4.79 (4.07, 5.72)	4.77 (4.07, 5.69)	4.97 (4.38, 5.92)	0.21
24-h urinary protein (g/day)	1.43 (0.70, 2.95)	1.39 (0.69, 2.87)	1.99 (1.10, 4.47)	0.01
Serum C3 (g/L)	0.88 (0.78, 1.01)	0.88 (0.78, 1.01)	0.92 (0.77, 1.02)	0.38
Serum C4 (g/L)	0.21 (0.17, 0.25)	0.21 (0.17, 0.25)	0.21 (0.17, 0.27)	0.68
ACEI/ARB (*n*, %)	433 (67.45%)	406 (67.55%)	27 (65.85%)	0.81
Immunosuppression therapy (*n*, %)	378 (58.88%)	355 (59.07%)	23 (56.10%)	0.70
Median follow-up period (months)	43.18 (31.13, 59.23)	43.23 (31.63, 59.73)	39.43 (26.00, 56.17)	0.21
Oxford classification				
Mesangial hypercellularity (M1)	390 (60.75%)	359 (59.73%)	31 (75.61%)	0.04
Endocapillary hypercellularity (E1)	46 (7.17%)	39 (6.49%)	7 (17.07%)	0.01
Segmental glomerulosclerosis (S1)	330 (51.40%)	313 (52.08%)	17 (41.46%)	0.19
Tubular atrophy/interstitial fibrosis				0.47
T1	115 (17.91%)	106 (17.64%)	9 (21.95%)	
T2	42 (6.54%)	41 (6.82%)	1 (2.44%)	
Cellular or fibrocellular crescents				0.09
C1	134 (20.87%)	124 (20.63%)	10 (24.39%)	
C2	16 (2.49%)	13 (2.16%)	3 (7.32%)	
Global sclerosis				0.28
G1	140 (20.81%)	133 (22.13%)	7 (17.07%)	
G2	48 (7.48%)	47 (7.82%)	1 (2.44%)	
Immune complex deposition				
IgG	77 (11.99%)	63 (10.48%)	14 (34.15%)	<0.01
IgM	305 (47.51%)	267 (44.43%)	38 (92.68%)	<0.01
C3	506 (78.82%)	470 (78.20%)	36 (87.80%)	0.15
C1q	80 (12.46%)	55 (9.15%)	25 (60.96%)	<0.01

### Pathological characteristics

On the immunofluorescent microscope of renal biopsy, approximately 47.51% of patients showed combined IgM deposition. A majority of patients (*n* = 506, 78.82%) displayed C3 deposition in the mesangium. Mesangial deposits of IgG and C1q were found in 77 and 80 patients, respectively. The immunopathological phenotype showed that the rates of IgG, IgM, and C1q deposition in the mesangium were significantly higher in the C4 positive group (Table 1, *p* < 0.05). Under the light microscope, patients with C4 deposition showed significantly higher rates of mesangial hypercellularity and endocapillary hypercellularity (*p* < 0.05). A higher rate of global sclerosis was found in the C4 negative group, while the difference between the two groups was not significant (*p* = 0.28). The subgroup analysis was shown in Table S1.

### Renal survival

**Figure 1.**
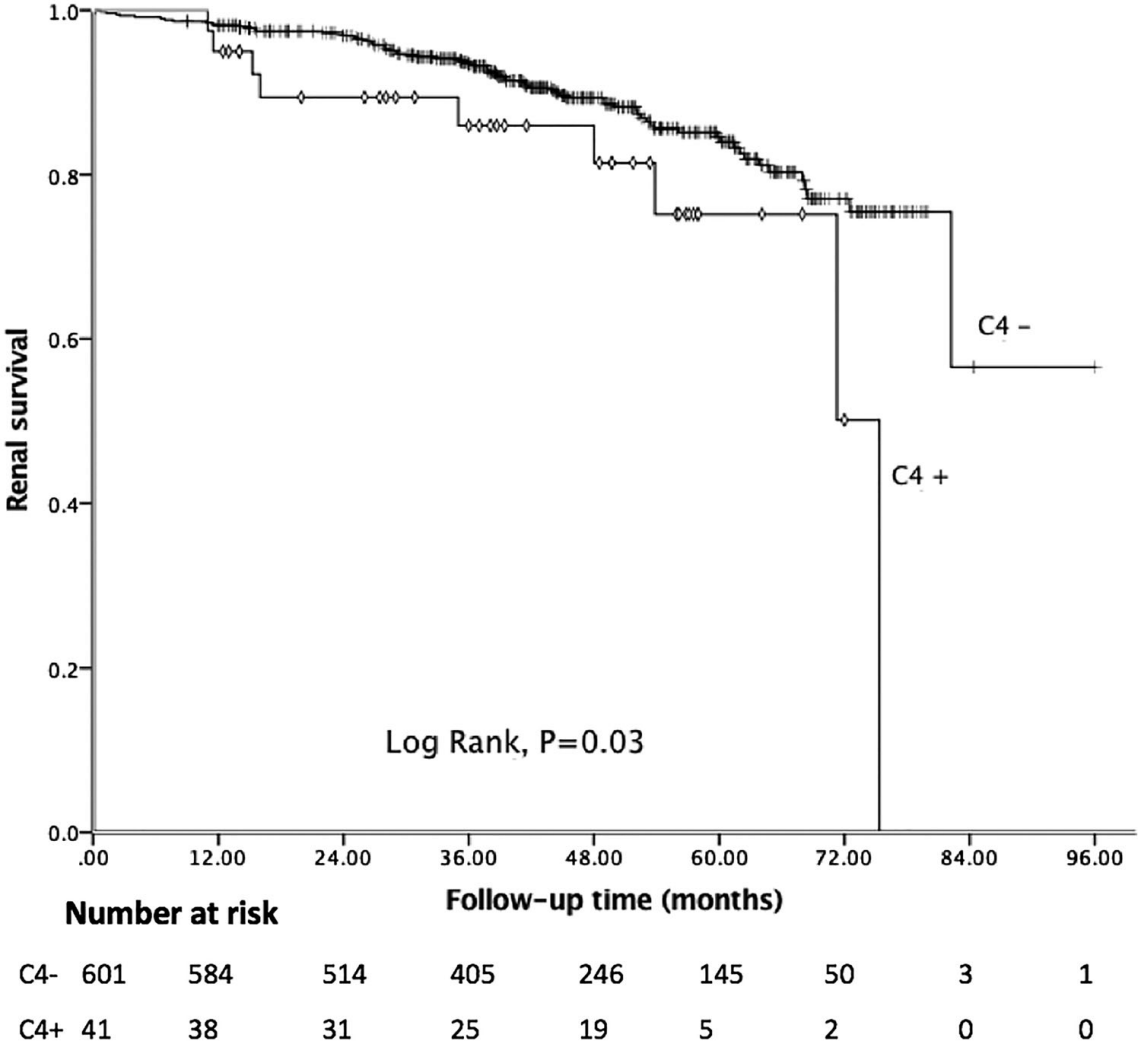
Kaplan–Meier analysis for the survival probability of the composite endpoint by C4 groups.

**Figure 2.**
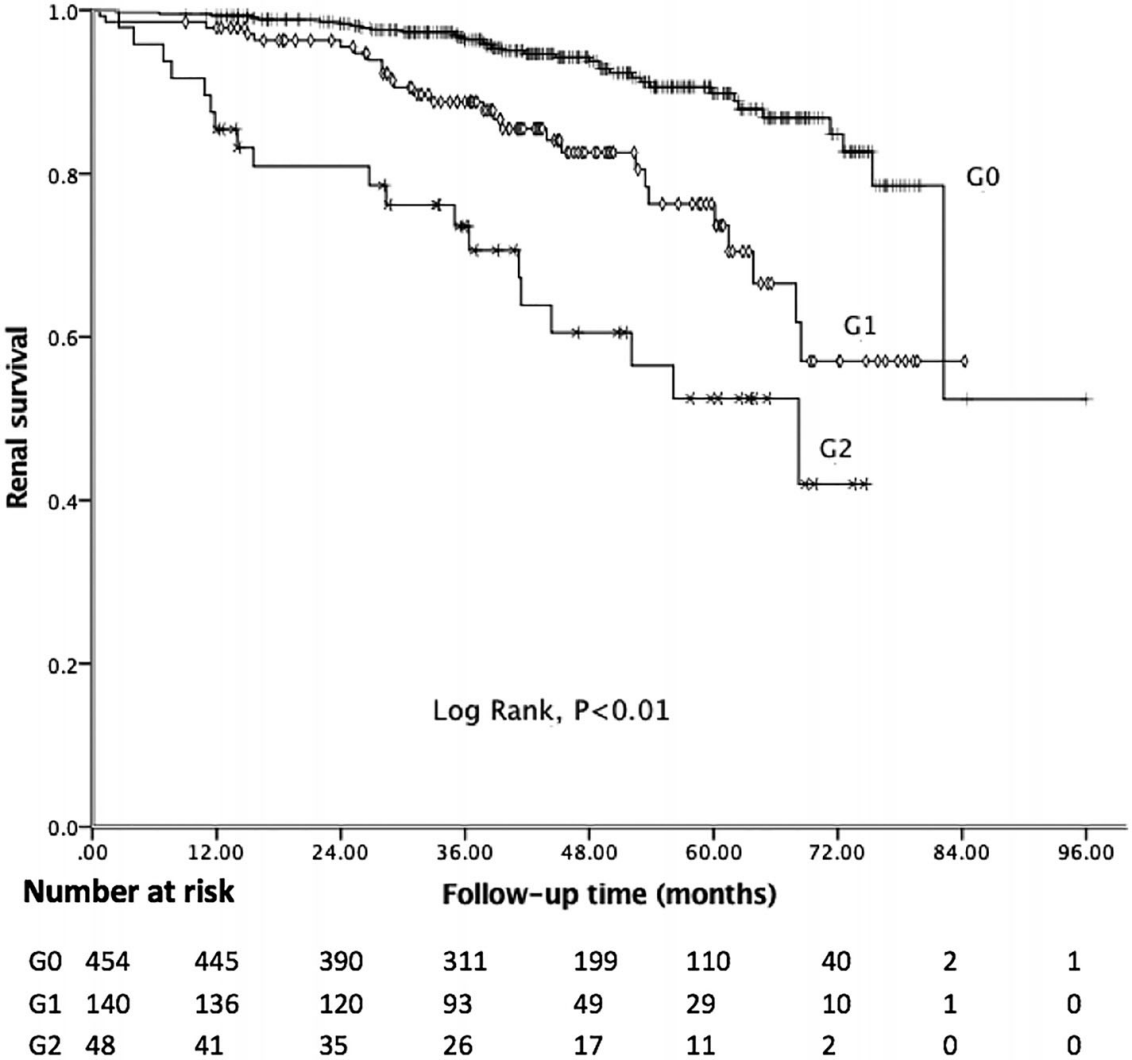
Kaplan–Meier analysis for the survival probability of the composite endpoint by different G-scores.

**Figure 3.**
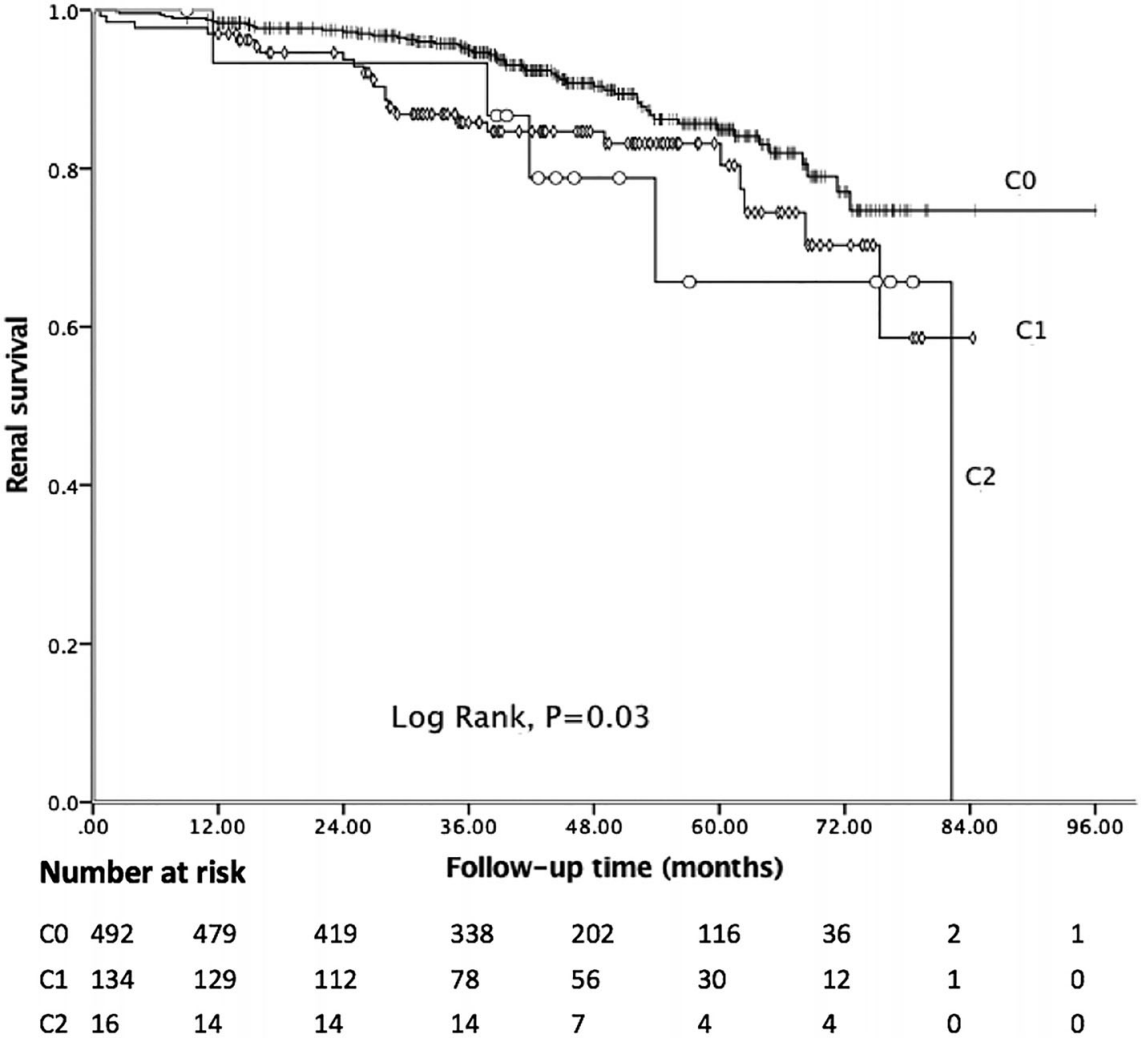
Kaplan–Meier analysis for the survival probability of the composite endpoint by different C-scores.

In the present study, the median follow-up period was 43.18 months, and 81 (12.62%) patients achieved the composite endpoint, in which 43 patients progressed to ESRD. Among 41 patients with C4 deposition, nine reached the endpoint. The Kaplan–Meier curve showed 5-year renal survival rate was 89.41%. As shown in Figure 1, patients with C4 deposition showed poor renal outcomes. Five-year renal survival rate was 82.93% in the C4 positive group compared with 89.85% in the C4 negative group (Log rank: *p* = 0.03). Considering global sclerosis, different G-scores were also related to poor renal survival (Log rank: *p* < 0.01, Figure 2). Figure 3 displays the Kaplan–Meier analysis for the probability of the composite endpoint in patients with different C-scores (Log rank: *p* = 0.03) and indicates that formation of cellular or fibrocellular crescents is related to renal outcomes.

### Related risk factors

The correlations between clinicopathological features and the composite endpoint were analyzed by Cox regression models as well. In the univariate analysis, several factors were significantly associated with an increased risk for disease progression, besides three above-mentioned variables that include C4 deposition, G-scores, and C-scores (see Table 2). The significant factors were male, hypertension, decreased hemoglobin, renal impairment, lower albumin, increased uric acid and triglyceride, more 24 h urinary protein, decreased C3 level and increased C4 level, mesangial hypercellularity, tubular atrophy or interstitial fibrosis, and C1q deposition.

**Table 2.** Univariate Cox regression analyses for the risk factors of renal survival.

Variable	Univariate analysis	*p*-value
	HR (95% CI)	
Gender (male vs. female)	1.58 (1.03, 2.48)	0.04
Age (years)	1.00 (0.98, 1.02)	0.74
Hypertension	2.14 (1.39, 3.32)	<0.01
Hemoglobin (g/L)	0.99 (0.98, 1.00)	0.04
Albumin (g/L)	0.95 (0.92, 0.98)	<0.01
Serum creatinine (µmol/L)	1.01 (1.01, 1.02)	<0.01
eGFR (ml/min/1.73m^2^)	0.97 (0.96, 0.98)	<0.01
Uric acid (µmol/L)	1.01 (1.00, 1.01)	<0.01
Triglyceride (mmol/L)	1.24 (1.13, 1.36)	<0.01
Cholesterol (mmol/L)	1.09 (0.97, 1.24)	0.16
24-h urinary protein (g/day)	1.08 (1.05, 1.12)	<0.01
Serum C3 (g/L)	0.20 (0.05, 0.74)	0.02
Serum C4 (g/L)	10.58 (1.15, 97.60)	0.04
Serum C3/C4	0.66 (0.51, 0.85)	<0.01
Immunosuppression therapy	1.30 (0.82, 2.07)	0.26
Oxford classification		
M1	1.65 (1.02, 2.68)	0.04
E1	0.99 (0.45, 2.16)	0.98
S1	1.28 (0.82, 2.00)	0.27
T1 vs. T0	3.00 (1.82, 4.96)	<0.01
T2 vs. T0	6.34 (3.60, 11.15)	<0.01
C1 vs. C0	1.72 (1.06, 2.79)	0.03
C2 vs. C0	2.21 (0.87, 5.61)	0.10
Global sclerosis		
G1 vs. G0	2.78 (1.68, 4.59)	<0.01
G2 vs. G0	6.35 (3.63, 11.13)	<0.01
Immune complex deposition		
C3 (+ vs. −)	1.47 (0.82, 2.61)	0.19
C4 (+ vs. −)	2.13 (1.07, 4.27)	0.03
C1q (+ vs. −)	1.78 (1.05, 3.01)	0.03

HR: hazard ratio; CI: confidence interval.

These variables were further analyzed by a multivariate Cox regression model. C4 deposition (HR = 3.22, 95%CI = 1.51–6.87, *p* < 0.01, Table 3), and global sclerosis (G1 vs. G0, HR = 1.90, 95%CI = 1.02–3.52, *p* = 0.04; G2 vs. G0, HR = 3.72, 95%CI = 1.98–7.00, *p* < 0.01) remained as significant predictors of IgAN progression. The other independent risk factors were male (HR = 1.80, 95%CI = 1.10–2.97, *p* = 0.02), serum creatinine (HR = 1.01, 95%CI = 1.00–1.01, *p* < 0.01), triglyceride (HR = 1.17, 95%CI = 1.01–1.35, *p* = 0.04), proteinuria (HR = 1.07, 95%CI = 1.01–1.13, *p* = 0.02), serum C3 level (HR = 0.05, 95%CI = 0.01–0.25, *p* < 0.01), and serum C4 level (HR = 99.59, 95%CI = 8.69–1140.89, *p* < 0.01).

**Table 3.** Multivariate Cox regression analyses for the risk factors of renal survival.

Variable	Multivariate analysis	*p*-value
	HR (95% CI)	
Gender (male vs. female)	1.80 (1.10, 2.97)	0.02
Serum creatinine (µmol/L)	1.01 (1.00, 1.01)	<0.01
Triglyceride (mmol/L)	1.17 (1.01, 1.35)	0.04
24-h urinary protein (g/day)	1.07 (1.01, 1.13)	0.02
Serum C3 (g/L)	0.05 (0.01, 0.25)	<0.01
Serum C4 (g/L)	99.59 (8.69, 1140.89)	<0.01
Global sclerosis		
G1 vs. G0	1.90 (1.02, 3.52)	0.04
G2 vs. G0	3.72 (1.98, 7.00)	<0.01
Immune complex deposition		
C4 (+ vs. −)	3.22 (1.51, 6.87)	<0.01

HR: hazard ratio; CI: confidence interval.

## Discussion

IgAN is recognized as a highly variable course ranging from a benign condition to progression to ESRD. Although previous studies have identified multiple risk factors influencing IgAN progression, including male, hypertension, renal impairment, urinary protein excretion over 1 g/d, and severe pathology [16,17], increasingly researchers are focusing on the role of complement [11,18]. However, the predictive value of serum complement level and complement deposition in prognosis of IgAN is still controversial.

In our study, increased serum C4 level was significantly associated with the poor renal survival in univariate and multivariate analyses. Similarly, Pan et al. found that increased C4 level was related to prognosis of IgAN, and was significantly associated with eGFR, segmental glomerulosclerosis, and tubular lesions [11]. Bi et al. [19] also confirmed that a higher serum C4 was a useful predictor in IgAN progression. They found patients with C4 deposition had higher serum C4 level, whereas no significant association was found between C4 deposition and prognosis. In contrast, Zhu et al. found that serum C4 level was positively associated with MAP, proteinuria, serum IgA, serum C3, and ST-scores, whereas it was negatively correlated with eGFR, serum IgM, IgG, IgM, and C3 deposition. The report of Zhu et al. indicated that patients with a low C4 level exhibited less severe renal injury and might have poor outcome [18]. Consistent with a previous study by Min et al. [20], we found that a decreased C3/C4 ratio was associated with poor renal outcomes. Min et al. also detected that patients with a lower C3/C4 ratio could benefit from aggressive immunosuppressive therapies [20]. Although C3/C4 ratio was not an independent risk factor in our report, our findings suggested that serum complement level was an important predictor in IgAN progression. We also found that a decreased C3 level was associated with IgAN progression. Pan et al. [11] reported that C3 level was an independent risk factor for poor renal outcomes in IgAN patients, while there was no significant association between C3 level and MEST-C scores or baseline eGFR. Similarly, a Korean study by Kim et al. [12] showed that low C3 level and mesangial C3 deposition were associated with progression. Conversely, Komatsu et al. [21] found patients with high IgA/C3 level had a significantly poorer renal outcome, suggesting that IgA/C3 ratio rather than C3 level alone could serve as a more appropriate marker in IgAN progression.

Interestingly, we detected glomerular C4 deposition was an independent novel predictor of poor prognosis in IgAN. In addition, we found that patients with mesangial C4 deposition showed higher rates of IgG, IgM, and C1q co-deposition. However, previous studies paid more attention to C4d deposition, which is a degradation product of the complement cascade [13,22]. Maeng et al. [22] showed that glomerular C4d deposition was significantly correlated with albuminuria, while tubular C4d deposition was significantly correlated with a higher grade of WHO classification for IgAN, and they speculated that patients with positive C4d staining might be related to renal damage. A multicenter retrospective study in Spain by Espinosa et al. [13] showed that C4d positive IgAN patients (38.5%) had a higher rate of hypertension, lower eGFR, and more proteinuria. Espinosa et al. [13] also found that mesangial C4d staining was an independent risk factor for progression to ESRD in IgAN patients. However, they did not consider the effect of intensity of C4d deposition on the IgAN prognosis. In our study, only 6.39% of IgAN patients were found C4 deposition, which was lower than C4d deposition rate. We used rabbit anti-human C4c as the antibody that was confirmed to be C4c-specific, as it showed no cross-reactivity with native C4, C4b, iC4b, or C4d [23]. The C4c is more common in circulation, and its binding ability in renal tissue is inferior to C4d. The activation of C4 involves in classical pathway and lectin pathway, which can lead to deposition of mannan-binding lectin (MBL) and C1q. Roos et al. [24] showed MBL deposition was related to more severe renal disease, and C4 or C4d deposits were correlated with co-deposition of MBL-associated serine proteases, proving that lectin pathway also participates in IgAN. Liu et al. [25] confirmed the role of glomerular MBL deposition in Chinese IgAN cohort, they found patients with MBL deposition had a lower renal remission rate. In our cohort, 12.46% of patients showed C1q deposition, and it was associated with composite endpoint in univariate analysis. Glomerular C1q deposition might be attributed to some unknown secondary causes which couldn’t be identified by our current detection methods. These patients need long-term and careful follow-up. Lee et al. [26] reported 8.1% of IgAN patients showed mesangial C1q deposition, they had severer clinical and pathologic features than C1q-negative patients. C1q as the initial factor of classical pathway can be activated by immune complexes, and further lead to glomerular injury and aggravation of pathological changes [27]. It seems that activation of the classical pathway might not be specific for IgAN but rather occurred in highly damaged kidneys [9]. All these studies revealed that multiple pathways of complement activation are involved in pathogenesis of IgAN. However, whether classical pathway is involved in IgAN pathogenesis is still unclear, further studies are needed to evaluate the efficacy of complement and to elaborate the role of complement activation in IgAN prognosis.

Consistent with previous studies [2,16], we found male, hypertension, decreased eGFR, and proteinuria were risk factors for renal outcomes. Similarly, Clinical Decision Support System for IgAN also suggested that age, gender, hypertension, serum creatinine, proteinuria, and histological grade were predictors for renal outcomes. Liu et al. [28] fitted a random forest model to predict ESRD for IgAN patients and found that C3 staining, MEST-C scores, and eGFR could convey additional information, and a better interpretative model could provide greater insights into prediction regarding ESRD in IgAN patients.

For histopathologic features, we found that global sclerosis, a chronic pathological change, was related to IgAN progression. Coincidentally, Kawamura et al. [3] found that global sclerosis could predict progression to ESRD in IgAN patients either requiring dialysis within 5 years or requiring dialysis within 5–10 years, indicating that global sclerosis was related to long-term prognosis. Chen et al. [29] also included global sclerosis in a prediction model and built the Nanjing IgAN Risk Stratification System in China. Thus, it is reasonable to add global sclerosis in the histological score system of IgAN.

We identified cellular or fibrocellular crescents and tubular atrophy or intestinal fibrosis were correlated with the composite endpoint in univariate analysis. In the original Oxford cohort, cellular or fibrocellular crescents were not significantly associated with renal survival, which may result from entry criteria [30]. Haas et al. [31] investigated a large cohort pooled from four retrospective studies and found that crescents were associated with poor renal outcomes. Both their study and our study ignored the influence of fibrous crescents. However, Walsh et al. [32] found that fibrous crescents were independent risk factors for progressive IgAN. Therefore, the effect of crescents on prognosis still needs more studies to validate.

Several limitations of our study need more considerations. First, the applicability of our findings to other ethnic groups needs further validation in other cohorts, as our study is a retrospective study limited to Chinese population. Second, the follow-up time of 5 years in this study was relatively short considering that IgAN progresses slowly. Third, our study only included immune complex deposition at the mesangium and ignored the effect of deposition at other positions and the intensity of deposition. Fourth, as only a few patients presented C4 deposition in this report, the results need further confirmation by a larger dataset. Therefore, large multiple-cohort studies are needed to identify the risk factors affecting IgAN progression and to determine the role of C4 in prognosis of IgAN. Further researches about C4 activation in IgAN are also required.

## Conclusion

In conclusion, mesangial C4 deposition and glomerulosclerosis are independent risk factors in IgAN progression. Our findings strongly suggest that complement activation is associated with prognosis of IgAN.

## Supplementary Material

Supplemental Material

Supplemental Material
