# Supplementary material for: Glomerular C4 deposition and glomerulosclerosis predict worse renal outcomes in Chinese patients with IgA nephropathy
Source: Ren Fail. 2020 Jul 14;42(1):629–37. doi: 10.1080/0886022X.2020.1786400 (PMC7470092; doi:10.1080/0886022X.2020.1786400)
Supplement: Supplemental Material [file IRNF_A_1786400_SM0691.pdf]

Table S1. Subgroup analysis of baseline clinical characteristics and histopathologic features of 642 IgAN patients.

Characteristics	C4+C1q+ (n=25)	C4+C1q- (n=16)	C4-C1q- (n=546)	C4-C1q+(n=55)	*P*-value
Male (n, %)	9 (36.00%)	5 (31.25%)	247 (45.24%)	20 (36.36%)	0.34
Age (years)	31.35 (28.23, 42.66)	39.21 (29.63, 46.71)	33.36 (26.55, 42.75)	33.24 (27.34, 41.00)	0.44
MAP (mmHg)	101.33 (94.67, 116.67)	98.83 (89.33, 123.17)	96.67 (88.67, 106.83)	105.00 (97.00, 119.33)	<0.01
Hypertension (n, %)	10 (40.00%)	7 (43.75%)	178 (32.60%)	27 (49.09%)	0.07
Hemoglobin (g/L)	134.00 (124.00, 141.00)	131.00 (116.50, 142.50)	132.00 (120.00, 148.00)	134.00 (124.00, 153.00)	0.64
Albumin (g/L)	38.60 (33.10, 41.70)	35.40 (30.40, 38.30)	39.40 (35.75, 42.80)	38.00 (32.10, 42.10)	0.01
Serum creatinine (μmol/L)	91.00 (70.00, 142.10)	81.00 (53.85, 144.75)	86.00 (67.70, 111.90)	110.00 (69.39, 127.00)	0.68
eGFR (ml/min/1.73m^2)	72.61 (38.43, 110.30)	85.93 (63.32, 113.11)	86.11 (63.26, 110.07)	66.42 (50.09, 103.00)	0.31
Uric acid (μmol/L)	389.00 (324.00, 426.70)	338.00 (266.80, 437.50)	365.00 (302.50, 440.95)	392.00 (289.00, 466.60)	0.92
Triglyceride (mmol/L)	1.52 (1.17, 2.27)	2.16 (1.08, 3.36)	1.51 (1.04, 2.23)	1.75 (1.33, 2.25)	0.15
Cholesterol (mmol/L)	4.76 (4.16, 5.54)	5.55 (4.62, 6.04)	4.76 (4.06, 5.61)	5.18 (4.48, 6.12)	0.08
24h urinary protein (g/day)	1.17 (0.94, 2.10)	4.28 (1.92, 6.63)	1.33 (0.68, 2.84)	2.36 (0.84, 3.50)	<0.01
Serum C3 (g/L)	0.88 (0.77, 0.96)	0.93 (0.87, 1.02)	0.88 (0.78, 1.01)	0.86 (0.76, 0.94)	0.30
Serum C4 (g/L)	0.20 (0.17, 0.22)	0.25 (0.18, 0.27)	0.21 (0.17, 0.25)	0.21 (0.16, 0.26)	0.27
ACEI/ARB (n, %)	18 (72.00%)	9 (56.25%)	268 (49.08%)	38 (69.09%)	0.75
Immunosuppression therapy (n, %)	14 (56.00%)	9 (56.25%)	319 (58.42%)	36 (65.45%)	0.77
Oxford classification					
Mesangial hypercellularity (M1)	19 (76.00%)	12 (75.00%)	327 (59.89%)	32 (58.18%)	0.25
Endocapillary hypercellularity (E1)	4 (16.00%)	3 (18.75%)	33 (6.04%)	6 (10.91%)	0.04
Segmental glomerulosclerosis (S1)	9 (36.00%)	8 (50.00%)	293 (53.66%)	20 (36.36%)	0.04
Tubular atrophy/interstitial fibrosis					0.75
T1	5 (20.00%)	4 (25.00%)	94 (17.22%)	12 (21.82%)	
T2	1 (4.00%)	0 (0.00%)	39 (7.14%)	2 (3.64%)	
Cellular or fibrocellular crescents					0.22
C1	6 (24.00%)	4 (25.00%)	108 (19.78%)	16 (29.09%)	
C2	2 (8.00%)	1 (6.25%)	11 (2.01%)	2 (3.64%)	
Global sclerosis					0.21
G1	3 (12.00%)	4 (25.00%)	119 (21.79%)	14 (25.45%)	
G2	1 (4.00%)	0 (0.00%)	39 (7.14%)	8 (14.55%)	
Immune complex deposition					
IgG	7 (28.00%)	7 (43.75%)	54 (9.89%)	9 (16.36%)	<0.01
IgM	25 (100.00%)	13 (81.25%)	220 (40.29%)	47 (85.45%)	<0.01
C3	23 (92.00%)	13 (81.25%)	427 (78.21%)	43 (78.18%)	0.42
