# Supplementary figures and images for: Glomerular C4 deposition and glomerulosclerosis predict worse renal outcomes in Chinese patients with IgA nephropathy
Source: Ren Fail. 2020 Jul 14;42(1):629–37. doi: 10.1080/0886022X.2020.1786400 (PMC7470092; doi:10.1080/0886022X.2020.1786400)

Figure S1. Enrollment and follow-up of study participants.

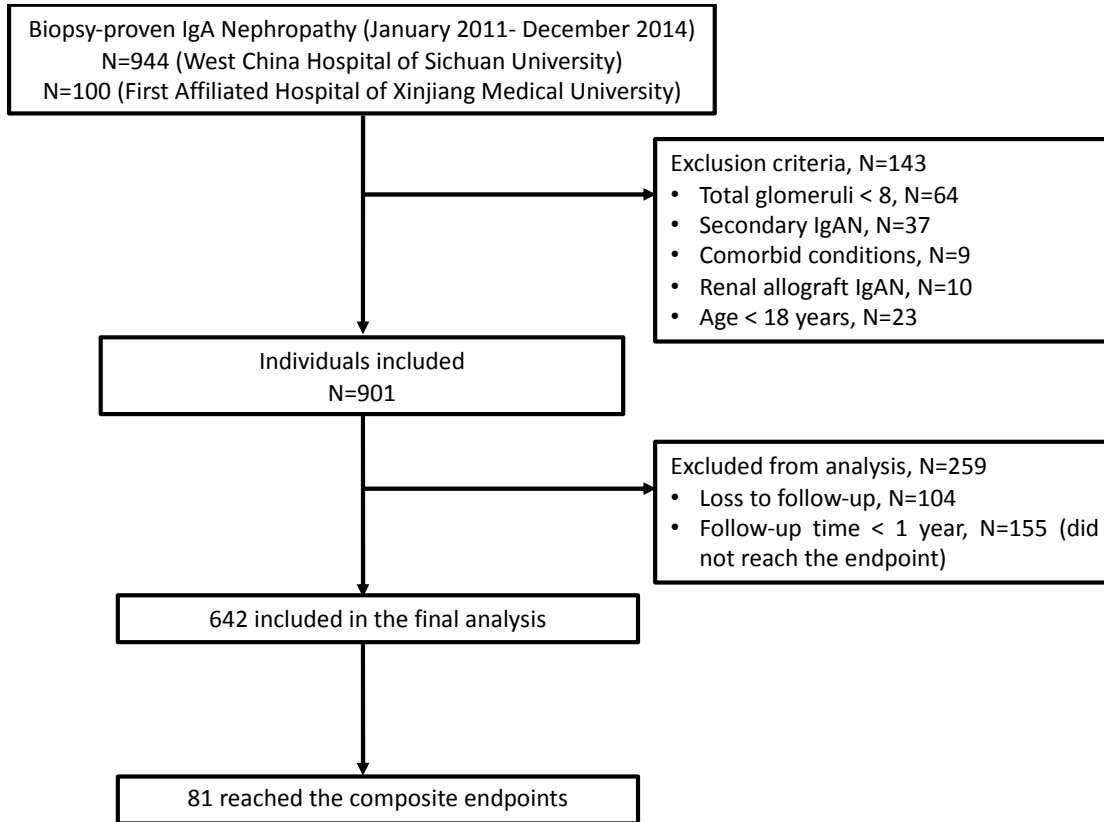

Supplement: Supplemental Material [file IRNF_A_1786400_SM0678.pdf]
